# Triweekly administration of parathyroid hormone (1–34) accelerates bone healing in a rat refractory fracture model

**DOI:** 10.1186/s12891-017-1917-2

**Published:** 2017-12-21

**Authors:** Yohei Kumabe, Sang Yang Lee, Takahiro Waki, Takashi Iwakura, Shunsuke Takahara, Michio Arakura, Yu Kuroiwa, Tomoaki Fukui, Tomoyuki Matsumoto, Takehiko Matsushita, Kotaro Nishida, Ryosuke Kuroda, Takahiro Niikura

**Affiliations:** 1 Department of Orthopaedic Surgery, Kobe University Graduate School of Medicine, 7-5-1 Kusunoki-cho, Chuo-ku, Kobe, 650-0017 Japan; 2 Department of Orthopaedic Surgery, Showa University School of Medicine, 1-5-8 Hatanodai, Shinagawa-ku, Tokyo, 142-8666 Japan

**Keywords:** Parathyroid hormone (1–34), Teriparatide, Fracture healing, Delayed union, Nonunion

## Abstract

**Background:**

Some reports have shown that intermittent parathyroid hormone (PTH) (1–34) treatment for patients with delayed union or nonunion have led to successful healing. In this study, we investigated whether systemic intermittent administration of PTH (1–34) has a beneficial effect on bone healing in a rat refractory fracture model.

**Methods:**

We created a refractory femoral fracture model in 32 rats with periosteal cauterization that leads to atrophic nonunion at 8 weeks after surgery. Half the rats received subcutaneous intermittent human PTH (1–34) injections at a dosage of 100 μg/kg, thrice a week for 8 weeks. The other half received the vehicle only. At 8 weeks after fracture, radiographic, histological and mechanical assessments were performed.

**Results:**

Radiographic assessments showed that the union rate was significantly higher in the PTH group than in the control group (*P* < 0.05). The degree of fracture repair as scored using the Allen grading system in histological assessment was significantly greater in the PTH group than in the control group (*P* < 0.05). The ultimate stress and stiffness measurements were significantly greater in the PTH group than in the control group (*p* < 0.05).

**Conclusions:**

We demonstrated that triweekly administration of PTH (1–34) increased union rate and accelerated bone healing in a rat refractory fracture model, suggesting that systemic administration of PTH (1–34) could become a novel and useful therapy for accelerating fracture healing in patients at high risk of delayed union or nonunion.

**Electronic supplementary material:**

The online version of this article (10.1186/s12891-017-1917-2) contains supplementary material, which is available to authorized users.

## Background

Approximately 5% of fractures are estimated to fail to heal and result in delayed union or nonunion [1]. Nonunion represents a challenge for the surgeon, the patient, and the healthcare system, creating a significant drain on a country’s resources and causing serious problems in a patient’s quality of life [2]. It has been reported that the estimated total cost of treatment for nonunion ranges between £7000 and £79,000 per person [3]. Treatment for nonunion usually requires multiple surgical procedures and prolonged hospitalization, with frequently unrewarding results. Therefore, the establishment of a therapeutic strategy for enhancing bone healing in cases of delayed union or nonunion, or in cases at high risk thereof is clinically warranted.

Parathyroid hormone (PTH) (1–34), also known as teriparatide, is a recombinant fragment of PTH that has an identical sequence to the biologically active region (the 34 N-terminal amino acids) of the full 84-amino acid PTH. The fundamental effect of PTH on bone is the enhancement of bone turnover through the stimulation of both osteoblast-mediated bone formation and osteoclast-mediated bone resorption. Intermittent exposure to PTH increases bone formation over resorption, thus causing an increase in net bone mass, while continuous infusion of PTH increases bone resorption over formation, thus causing a net loss of bone mass [4, 5]. Intermittent PTH therapy has been associated with the expansion of osteoblast and osteoprogenitor cell populations, with this expansion thought to be one of the major mechanisms through which PTH is able to favor bone formation [6]. Intermittent daily administration of PTH (1–34) has been proven to increase bone mineral density and decrease the risk of vertebral and non-vertebral fractures in osteoporotic patients [7].

Growing evidence suggests that intermittent daily PTH treatment can enhance fracture healing in several animal models and in patients in clinical trials [8–17]. Andreassen et al. investigated the efficacy of intermittent PTH (1–34) therapy (60 or 200 μg/kg/day) in a rat tibial fracture model, demonstrating increased strength at a fracture site and callus quality in both dose groups [8]. In a clinical trial, Aspenberg et al. showed that PTH (1–34) at a dosage of 20 μg/day improved early callus formation and shortened the time to radiological healing in a randomized control trial in 102 postmenopausal women with a distal radius fracture that was treated non-operatively [12]. However, there have been some animal and clinical studies in which PTH (1–34) therapy was less effective. Bhandari et al. did not observe an effect of 20 μg/day PTH (1–34) for 6 months on consolidation of femoral neck fractures in 159 patients after 2 years [18]. Alkhiary et al. tested the efficacy of 5 and 30 μg/kg/day in a rat femoral fracture model [10]. Although biomechanical properties significantly increased in the high-dose group, the effect of low-dose treatment was similar to that of placebo on day 35.

Another important potential application for PTH (1–34) is in the setting of impaired fracture healing. Recently, some small case series and isolated case reports have shown that intermittent PTH (1–34) treatment for patients with delayed union or nonunion have led to successful healing, suggesting the drug could be effective as an adjuvant for impaired fracture healing [19]. However, to date, there have been no clinical trials on the use of PTH (1–34) to accelerate bone healing in delayed union and nonunion. In addition, limited data has described the role of PTH (1–34) on impaired fracture healing in experimental animals [16, 19].

Different frequencies of PTH (1–34) administration (e.g., daily versus weekly versus triweekly [20]) have been shown to have different effects on fracture healing and bone regeneration [20, 21]. Previous studies indicated that less frequent administration of PTH (1–34) might be a feasible protocol. Tsunori et al. compared the efficacy of once-daily (15 μg/kg), triweekly (35 μg/kg or 105 μg/kg), and once weekly (105 μg/kg) PTH (1–34) injections for bone regeneration in critical-size bone defects in the rat calvarium [20]. This study revealed that triweekly PTH (1–34) administration at a higher dose had the greatest effect on bone regeneration. However, no study has investigated the effect of less frequent intermittent administration of PTH (1–34) on refractory fractures or nonunion in experimental animals. In this study, we aimed to determine whether systemic triweekly administration of PTH (1–34) enhances bone healing in a rat refractory fracture model.

## Methods

### Animal model

Thirty-two twelve-week-old male Sprague-Dawley rats (CLEA, Tokyo, Japan) weighing 392.5 ± 7.2 g were used in this study. We created a reproducible refractory femoral fracture model with periosteal cauterization in the rats, which leads to atrophic nonunion at 8 weeks after surgery [22]. Briefly, a 1.25-mm diameter K-wire was inserted into the right femoral intramedullary canal in a retrograde fashion and a closed transverse femoral shaft fracture was created in the right femur using a 3-point bending apparatus with a drop weight [23, 24]. In order to produce nonunion, the fractured site was exposed and the periosteum was cauterized circumferentially for 2-mm on each side of the fracture.

### PTH treatment

Immediately after the procedure, half the rats (*n* = 16) received subcutaneous intermittent human PTH (1–34) (a generous gift from Asahi Kasei Pharma Corp., Tokyo, Japan) injections at a dosage of 100 μg/kg, thrice a week for 8 weeks (PTH group). The PTH was dissolved in saline with 0.1% rat serum albumin. The other half rats (*n* = 16) received the vehicle only (control group). At 8 weeks after fracture, all animals were euthanized by an overdose of pentobarbital sodium intraperitoneally for the following assessments.

### Radiographic assessment of fracture repair

At week 8, radiographs of the fractured limb from anteroposterior and lateral views were taken. Each callus, on the four cortices (two from antero-posterior and two from lateral views), was evaluated by four orthopedic surgeons blinded to the group. A bony union was defined when three of the four cortices were bridged and/or fracture lines disappeared completely [25, 26]. Nonunion was defined if neither side of the callus was bridged in these views [26].

### Micro-computed tomography (μ-CT) measurement

At week 8, the fractured femur was harvested and the intramedullary pins were removed from the bones. For quantification of callus formation, micro-computed tomography (μ-CT) imaging was performed on seven animals, which were randomly selected, in each group by using R_mCT2 FX (Rigaku Corp., Tokyo, Japan) [Additional file 1: Table S1]. After scanning, 3D reconstruction was performed using built-in software. The region of interest (ROI) was set at the area of callus healing and extended 3-mm proximally and distally to the fracture line, resulting in a ROI of 6-mm. The following parameters of the callus were calculated from the ROI using bone microstructure software (TRI/3D–BON-FCS64, Ratoc System Engineering, Tokyo, Japan): tissue mineral density (TMD), total callus volume (TV), callus bone mineral content (BMC), volumetric bone mineral density (vBMD = BMC/TV), and bone volume fraction (the ratio of bone volume (BV) to TV; BV/TV). Calibration of BMD was performed by scanning hydroxyapatite phantoms of known densities that were provided by the system manufacturer.

### Histological assessment

At week 8, the fractured femur was harvested from six animals, which were randomly selected, in each group (Additional file 1: Table S1). The femur was fixed in 4% paraformaldehyde, decalcified, and embedded in paraffin wax. Sagittal sections were prepared and stained with Safranin-O/Fast Green. The Allen score, which utilizes a five-point scale (grades 0 through 4) for grading fracture healing, was evaluated by four orthopedic surgeons blinded to the group [27]. The grading scale for histological assessment of fracture healing is as follows: grade 0, nonunion; grade 1, incomplete cartilaginous union (fibrous elements); grade 2, complete cartilaginous union; grade 3, incomplete bony union (small amount of cartilage present in callus); and grade 4, complete bony union.

### Mechanical assessment

At week 8, biomechanical assessment was performed on six animals, which were randomly selected, in each group [Additional file 1: Table S1]. Briefly, the fractured femur and the contralateral, intact femur were harvested and the intramedullary fixation pin was removed. A standardized 3-point bending test was performed with a load torsion and bending tester (MZ-500D, MZ-500S, Maruto Instrument Co., Ltd., Tokyo, Japan) [28]. The bending force was applied with the crosshead at a speed of 2-mm/min until rupture occurred. The ultimate stress (in N), extrinsic stiffness (in N/mm), and failure energy (in N/mm) were assessed. For each parameter, the ratio of value in the fractured femur to that in the intact femur in the same animal was calculated.

### Statistical analysis

All the quantitative data was presented as means ± standard deviations. Fisher’s exact test was used for the radiographic assessment of union and nonunion rates, and the Mann-Whitney U test was used for data from μ-CT measurement, histological assessment, and mechanical assessment. A *P*-value of <0.05 was set as statistically significant.

## Results

### Union rate

**Fig. 1:**
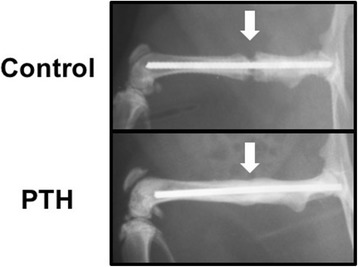
Radiographic evidence of fracture healing in the control group (upper panel) and the PTH group (lower panel) at week 8 after fracture. Representative radiographs including the fracture site (white arrow)

Eleven (68.8%) rats in the PTH group achieved fracture union compared with only 5 (31.3%) rats in the control group (Fig. 1 and Table 1; *P* < 0.05). In the control group, nonunion was observed in 9 (56.3%) rats, whereas in the PTH group nonunion was observed in only 3 (18.8%) rats (*P* < 0.05).

**Table 1:** Union and nonunion rate by radiographic assessment at week 8

	Control	PTH (1–34)
Union	5/16 (31.3%)	11/16 (68.8%)*
Nonunion	9/16 (56.3%)	3/16 (18.8%)*

*p < 0.05 compared with the control group

### μ-CT measurement

TMD of the callus was higher in the PTH group than in the control group (*p* < 0.05; 774.7 ± 56.5 vs 693.3 ± 41.8 mg/cm^3^; Table 2). vBMD of the callus was higher in the PTH group than in the control group (*p* < 0.05; 491.8 ± 75.4 vs 393.3 ± 35.6 mg/cm^3^). No significant difference in TV, BMC and BV/TV was observed between the PTH group and control group.

**Table 2:** Micro-computed tomography (μ-CT) measurement at week 8

Parameter	Control	PTH (1–34)
TMD (mg/cm^3^)	693.3 ± 41.8	774.7 ± 56.5*
TV (cm^3^)	0.2 ± 0.1	0.2 ± 0.0
BMC (mg)	87.5 ± 26.2	86.3 ± 19.6
vBMD (mg/cm^3^)	393.3 ± 35.6	491.8 ± 75.4*
BV/TV (%)	56.8 ± 5.1	63.2 ± 6.9

*TMD* tissue mineral density, *TV* total callus volume, *BMC* bone mineral content, *vBMD* volumetric bone mineral density (BMC/TV), *BV* bone volume, *BV/TV* bone volume fraction (the ratio of bone volume (BV) to TV)

*p < 0.05 compared with the control group

### Histological assessment

**Fig. 2:**
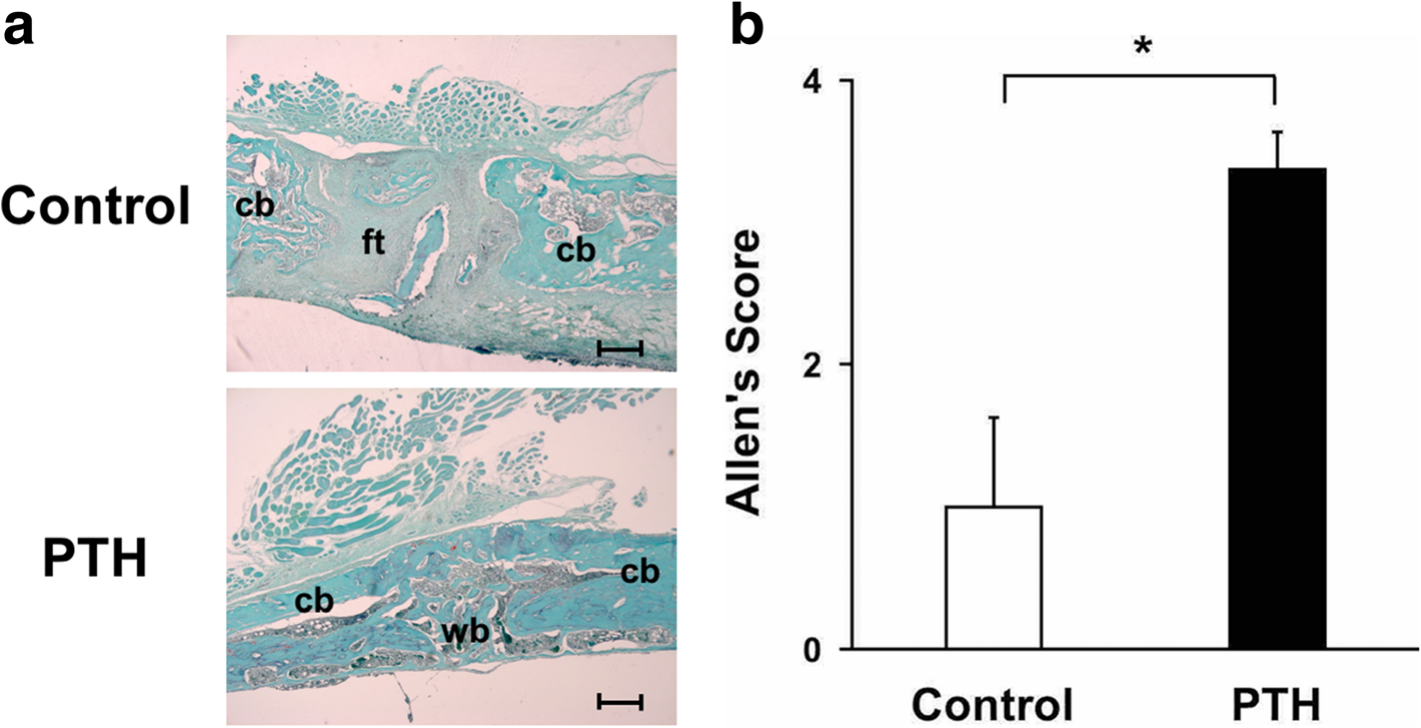
**a** Representative histological sections stained with Safranin-O/Fast Green. Cb = cortical bone, wb = woven bone, and ft. = fibrous tissue. Bar = 500 μm. **b** The degree of fracture healing as indicated by the mean Allen score at week 8 (*n* = 6 in each group) (*p < 0.05 in the indicated group)

Bridging at the fracture site by woven bone was observed at week 8 in the PTH group; this could hardly be found in the control group and the fracture site was filled with fibrous tissue (Fig. 2a). The Allen score in the PTH group was significantly greater than that in the control group (*P* < 0.05; 3.4 ± 0.5 vs 1.3 ± 1.6; Fig. 2b).

### Mechanical assessment

**Fig. 3:**
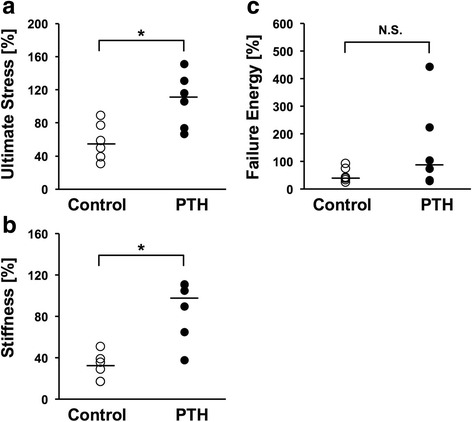
Biomechanical assessment of a fractured femur as assessed by the 3-point bending test at week 8 (*n* = 6 in each group). Values were normalized relative to the contralateral, intact femur (*p < 0.05 in the indicated group; N. S., no significance, medians are represented as horizontal lines)

The ultimate stress and stiffness measurements for the fractured femur (expressed as a percentage of value in the intact femur) were significantly greater in the PTH group than in the control group (*P* < 0.05; 107.5 ± 32.5% vs 57.5 ± 22.2%, *P* < 0.05, 86.6 ± 29.5% vs 31.5 ± 13.1%; respectively; Fig. 3a, b). There was no significant difference in failure energy between the two groups (151.6 ± 159.7% vs 52.0 ± 26.8%; Fig. 3c).

## Discussion

The failure of a fracture to unite represents an important problem in clinical practice. Off-label clinical use of PTH (1–34) is becoming an increasingly used option in challenging fractures and delayed union or nonunion, with successful early case reports [19]. However, the anecdotal literature on the use of PTH in promoting bone healing is only able to suggest its efficacy. This is the first study to elucidate the effect of systemic triweekly PTH (1–34) administration on bone healing using a rat refractory fracture model. In the current study, the union rate at week 8 as assessed by radiographs, and TMD and vBMD of the callus at week 8 as assessed by μ-CT, were significantly higher in PTH group than in the control group. Histological assessment revealed acceleration of bone healing in PTH-treated rats. Finally, ultimate stress and stiffness were significantly greater in the PTH group. Collectively, these results indicated that triweekly administration of PTH (1–34) accelerated bone healing and decreased the incidence of nonunion in rats.

To date, there have been only three studies on the use of PTH in experimental nonunion or refractory fracture models [29–31]. Lin et al. made a murine atrophic nonunion model by creating an open osteotomy in the midshaft femur, followed by distraction using a clip to maintain a fracture gap of 1.7-mm [30]. Mice were treated with daily injections of 30 μg/kg PTH (1–34) for 14 days. At week 6 after surgery, the PTH (1–34) treatment resulted in lower rates of nonunion measured both radiographically and histologically. In contrast, Tagil et al. did not find a beneficial effect from PTH (1–34) treatment in a rat refractory femoral fracture model [29]. They used a hypertrophic nonunion model that they generated by creating open osteotomies with circumferential periosteal stripping 15-mm proximally to and distally from the osteotomies. They then treated them with PTH (1–34) at a dose of 50 μg/kg, 5 days a week for 6 weeks. Although PTH (1–34) treatment produced significant increases in callus size and strength, it did not increase union rate at week 6 compared to the control group, which was assessed histologically and radiologically. In the study of Perez-Nunez et al., a model of atrophic nonunion with a 2-mm gap was created in a rat’s femur [31]. Rats were treated with PTH (1–84) at a dose of 30 μg/kg daily, 5 days a week for 12 weeks. There was no significant improvement, which was histologically and radiologically assessed, in bone healing at week 12 compared to the control group.

In our study, we used a rat refractory femoral fracture model, which had already been established by Kokubu et al. [22]. In our model, the periosteal was cauterized to simulate the periosteal disruption, which leads to atrophic nonunion at 8 weeks after fracture. This model simulates the altered biological and mechanical environment seen in high-energy fractures, in which there is often significant soft-tissue damage, including disruption of the periosteum. PTH (1–34) was administrated thrice a week at a dosage of 100 μg/kg. Using this model and treatment protocol, we were successfully able to achieve increased bony union rate, mineral density of callus, and strength. The discrepancy between previous studies and our results may be due to multiple factors, including differences in species, surgical technique, dosage, and administration schedules.

The dosing schedule of PTH (1–34) in the current study was set as thrice a week. Several studies have shown that PTH (1–34) administration three times a week significantly promoted bone healing [20, 32, 33]. Komatsubara et al. demonstrated that triweekly PTH (1–34) treatment accelerated the fracture healing process in a rat osteotomy model [32]. Consistent with the study, the present study revealed that administration of PTH (1–34) thrice a week enhanced bone healing in a rat refractory fracture model. In Japan, weekly administration of PTH (1–34) at a dosage of 56.5 μg is used to treat patients with osteoporosis. Nakamura et al. showed that a weekly subcutaneous injection of PTH (1–34) is effective at increasing BMD and reducing the risk of new vertebral fracture by 80% in older men and postmenopausal women [34]. Recently, some clinical case report suggested that weekly PTH (1–34) administration could be effective in promoting bone healing in delayed union or nonunion [35, 36]. Since the rate of bone turnover differs between humans and rats, a triweekly dosing frequency in rats was considered equivalent to a weekly dosing in humans [37]. Therefore, our findings indicate that a weekly injection of PTH (1–34) may have positive effects on bone healing in patients with delayed union or nonunion.

Several studies have identified potential mechanisms by which PTH (1–34) may enhance fracture healing [6, 15, 38]. PTH (1–34) may enhance fracture healing by two mechanisms: firstly by stimulating the differentiation of osteoblasts and secondly by promoting chondrocyte proliferation and differentiation and accelerating endochondral bone formation [9, 11, 39]. Nakajima et al. showed that PTH (1–34) promotes fracture healing by stimulating proliferation of osteoblastic progenitor cells, synthesis of bone matrix proteins, and osteoclastgenesis, thereby enhancing both callus formation and callus remodeling in a rat closed femoral fracture model [9]. Nakazawa et al. showed that PTH treatment increases the recruitment of mesenchymal cells into the chondrocyte lineage and proliferation of chondroprogenitor cells, resulting in the formation of a larger cartilaginous callus in the same fracture model [11]. Kakar et al. performed a comprehensive investigation of PTH (1–34) actions on endochondral bone formation using a mouse closed femoral fracture model [39]. In this study, PTH (1–34) mainly influenced the early phases of fracture healing through increased chondrogenic cell recruitment and accelerated chondrocyte maturation and mineralization, potentially through a mechanism that involved canonical Wnt signaling. Although the manner in which PTH (1–34) therapy affects the healing process in refractory fracture remains to be determined, it is possible that similar mechanisms may work.

The present study had some potential limitations. First, we did not explore the cellular mechanisms responsible for the results of PTH (1–34) therapy. PTH (1–34) promotes fracture healing by stimulating proliferation of osteoblastic progenitor cells, synthesis of bone matrix proteins, and osteoclastogenesis, thereby enhancing both callus formation and callus remodeling [9]. Thus, it would be of interest to assess osteoblasts and osteoclasts at the fracture site histologically, which may lead to understanding of the mechanism of enhanced bone healing in refractory fractured and nonunion with PTH (1–34) treatment. Secondly, the dosage of PTH (1–34) used in the study is much higher than the clinical dose (20 μg daily or 56.5 μg weekly) for human osteoporosis treatment. We chose the dose of 100 μg/kg based on our preliminary experiments and previous fracture-healing studies in rats that have shown beneficial effects [40]. The difference should be interpreted cautiously as rats and humans have important differences in skeletal and physiological properties and in their response to and metabolism of PTH (1–34) [10, 16]. However, a higher dose of PTH (1–34) than that for osteoporosis might be necessary to promote bone healing. Hence, the appropriate dosage and frequency for the treatment of nonunion needs to be defined in clinical studies because of difficulties in translating an animal dosage and frequency to human. These will be examined in future study.

## Conclusions

Systemic triweekly administration of PTH (1–34) increased union rate and accelerated bone healing in a rat refractory femoral fracture model. These findings suggest that intermittent administration of PTH (1–34) may become a novel and useful therapy for accelerating fracture healing in patients at high risk of delayed union or nonunion.

## Additional file

Additional file 1: Table S1. The table showing the performed assessments for each animal. (DOCX 18 kb)

## Abbreviations

BMC: Callus bone mineral content
BV: Bone volume
PTH: Parathyroid hormone
ROI: Region of interest
TMD: Tissue mineral density
TV: Total callus volume
VBMD: Volumetric bone mineral density
μ-CT: Micro-computed tomography
