# Supplementary material for: Triweekly administration of parathyroid hormone (1–34) accelerates bone healing in a rat refractory fracture model
Source: BMC Musculoskelet Disord. 2017 Dec 21;18:545. doi: 10.1186/s12891-017-1917-2 (PMC5740882; doi:10.1186/s12891-017-1917-2)
Supplement: Additional file 1: Table S1. — The table showing the performed assessments for each animal. (DOCX 18 kb) [file 12891_2017_1917_MOESM1_ESM.docx]

| **Sample no.** | **Radiographic Assessment** | **µ-CT**  **Measurement** | **Histological Assessment** | **Mechanical Assessment** |
| --- | --- | --- | --- | --- |
| **PTH-1** | + | + |  |  |
| **PTH-2** | + |  |  | + |
| **PTH-3** | + |  |  | + |
| **PTH-4** | + |  | + |  |
| **PTH-5** | + | + |  |  |
| **PTH-6** | + |  |  | + |
| **PTH-7** | + |  | + |  |
| **PTH-8** | + | + |  | + |
| **PTH-9** | + |  | + |  |
| **PTH-10** | + | + |  |  |
| **PTH-11** | + |  | + |  |
| **PTH-12** | + | + |  |  |
| **PTH-13** | + |  | + |  |
| **PTH-14** | + | + |  | + |
| **PTH-15** | + | + | + |  |
| **PTH-16** | + |  |  | + |
| **control-1** | + | + |  |  |
| **control-2** | + | + |  |  |
| **control-3** | + |  | + |  |
| **control-4** | + |  | + |  |
| **control-5** | + |  |  | + |
| **control-6** | + | + |  |  |
| **control-7** | + | + |  | + |
| **control-8** | + |  |  | + |
| **control-9** | + |  | + |  |
| **control-10** | + |  |  | + |
| **control-11** | + | + |  |  |
| **control-12** | + |  |  | + |
| **control-13** | + |  | + |  |
| **control-14** | + |  |  | + |
| **control-15** | + | + | + |  |
| **control-16** | + | + | + |  |
